# Visual and Rapid Diagnosis of *Neisseria gonorrhoeae* Using Loop-Mediated Isothermal Amplification Combined With a Polymer Nanoparticle–Based Biosensor in Clinical Application

**DOI:** 10.3389/fmolb.2021.702134

**Published:** 2021-07-21

**Authors:** Xu Chen, Qingxue Zhou, Xueli Wu, Shuoshi Wang, Rui Liu, Shilei Dong, Wei Yuan

**Affiliations:** ^1^ The Second Clinical College, Guizhou University of Traditional Chinese Medicine, Guiyang, China; ^2^ Central Laboratory of the Second Affiliated Hospital, Guizhou University of Traditional Chinese Medicine, Guiyang, China; ^3^ Clinical Laboratory, Hangzhou Women’s Hospital, Hangzhou, China; ^4^ Department of Clinical Laboratory, Zhejiang Hospital, Hangzhou, China; ^5^ Guizhou Provincial Center for Clinical Laboratory, Guiyang, China

**Keywords:** *Neisseria gonorrhoeae*, loop-mediated isothermal amplification, polymer nanoparticle–based biosensor, limit of detection, point-of-care testing

## Abstract

*Neisseria* *gonorrhoeae* is a host-adapted human pathogen that causes sexually transmitted gonorrhea and remains to be a serious global public health challenge, especially in low- and middle-income regions. It is vital to devise a reliable, simple, cost-saving, and easy-to-use assay for detecting the *N. gonorrhoeae* agent. In the current study, we firstly report a novel approach, loop-mediated isothermal amplification linked with a polymer nanoparticle–based biosensor (LAMP-PNB), that was used for identifying *N. gonorrhoeae* in clinical samples. The results showed that the LAMP primers based on the *orf1* gene were valid for development of the *N. gonorrhoeae*-LAMP-PNB assay. The detection system with optimal conditions could be performed at a fixed temperature of 64°C for 40 min. The whole process, including genomic DNA preparation (approximately 10 min), LAMP reaction (40 min), and PNB reporting (approximately 2 min), could be accomplished within 60 min. The limit of detection (LoD) of the *N. gonorrhoeae*-LAMP-PNB assay was 50 copies per test. The specificity of the current assay was 100%, and no cross-reactions to non–*N. gonorrhoeae* isolates were observed. These results confirmed that the *N. gonorrhoeae*-LAMP-PNB technique is a reliable, specific, sensitive, rapid, low-cost, and easy-to-use method for detecting gonococci isolates. More importantly, this assay has great potential to develop a point-of-care (POC) testing method in clinical practice, especially in resource-constrained regions.

## Introduction

*Neisseria gonorrhoeae*, a host-adapted human pathogen, is the causative agent of gonorrhea, which belongs to the most frequent sexually transmitted infections that remain one of the major global public health concerns (Quillin and Seifert, 2018; Hook and Bernstein, 2019). According to World Health Organization (WHO) estimations, there are around 87 million new infections worldwide each year (Rowley et al., 2019). Of these, the vast majority of gonococcal infections (>80 million) are in developing countries of Africa, Asia, and Latin America (World Health Organization, 2016; Rowley et al., 2019). *N. gonorrhoeae* infection can cause epididymo-orchitis and prostatitis in men and can lead to pelvic inflammatory disease, infertility, and ectopic pregnancy in women (Chan et al., 2016; Stevens and Criss, 2018). Maternal transmission to infants during birth can also bring about neonatal blindness and oropharyngeal infections (Vallely et al., 2021). In rare cases, *N. gonorrhoeae* could spread systemically, resulting in severe complications, such as septicemia, vasculitis, endocarditis, arthritis, and tenosynovitis (Lovett and Duncan, 2019). Because the various clinical symptoms of gonorrhea are largely not specific, and most gonococcal infections are in resource-constrained regions, developing a specific, sensitive, rapid, and cost-saving assay for the accurate identification of *N. gonorrhoeae* isolates is necessary for reducing ongoing gonorrhea transmission.

The traditional assay for identification of *N. gonorrhoeae* is based on cultivation. However, gonococcus is very demanding and fastidious for cultivation. The bacteria from swab collection samples should be immediately inoculated onto culture media, which require rigorous growth conditions; this method is time-consuming (24–48 h) and does not succeed equally well from every sample (Meyer and Buder, 2020). In recent decades, nucleic acid amplification technologies (NAATs), such as polymerase chain reaction (PCR), multiplex PCR, and real-time PCR, have been used for identifying *N. gonorrhoeae*. These methods are more sensitive, specific, and time-saving than cultivation and have been considered the primary methods to detect *N. gonorrhoeae* (O'Callaghan et al., 2010; Mahony et al., 1995; Whiley et al., 2002). Nevertheless, their use in many poor-resource settings is greatly limited due to high costs of experimental instruments and skilled personnel (Meyer and Buder, 2020). Hence, developing a low-cost, rapid, sensitive, specific, and simple diagnostic method for *N. gonorrhoeae* isolates is essential for follow-up treatment and management of gonorrhea patients.

To overcome the drawbacks of the PCR-based assay, loop-mediated isothermal amplification (LAMP) as a reliable, low-cost, sensitive, and rapid nucleic acid amplification technique was first devised by Notomi et al. (2000) and has been widely used to identify various pathogens, such as SARS-CoV-2, *Mycobacterium tuberculosis*, and *Brucella* (Li et al., 2019; Shete et al., 2019; Zhu et al., 2020). The LAMP method can efficiently amplify target genes at a constant temperature (usually 58–69°C) using *Bst* DNA polymerase within a short time (30–60 min) (Wong et al., 2018). The primer set consists of two outer primers (F3 and B3), two inner primers (FIP and BIP), and two loop primers (LF and LB) (Notomi et al., 2015). Previous studies have shown that the LAMP products could be analyzed with various technologies, such as colorimetric indicators (malachite green reagent), turbidimetry, and fluorescence dye (Chen et al., 2020). However, these detection techniques require special apparatus and reagents. To overcome these defects, a polymer nanoparticle–based biosensor (PNB), with specificity, sensitivity, visualization, good robustness, simple operation, and low limits of detection features, has been applied for detection of DNA and proteins in recent years (Quesada-González and Merkoçi, 2015).

**FIGURE 1:**
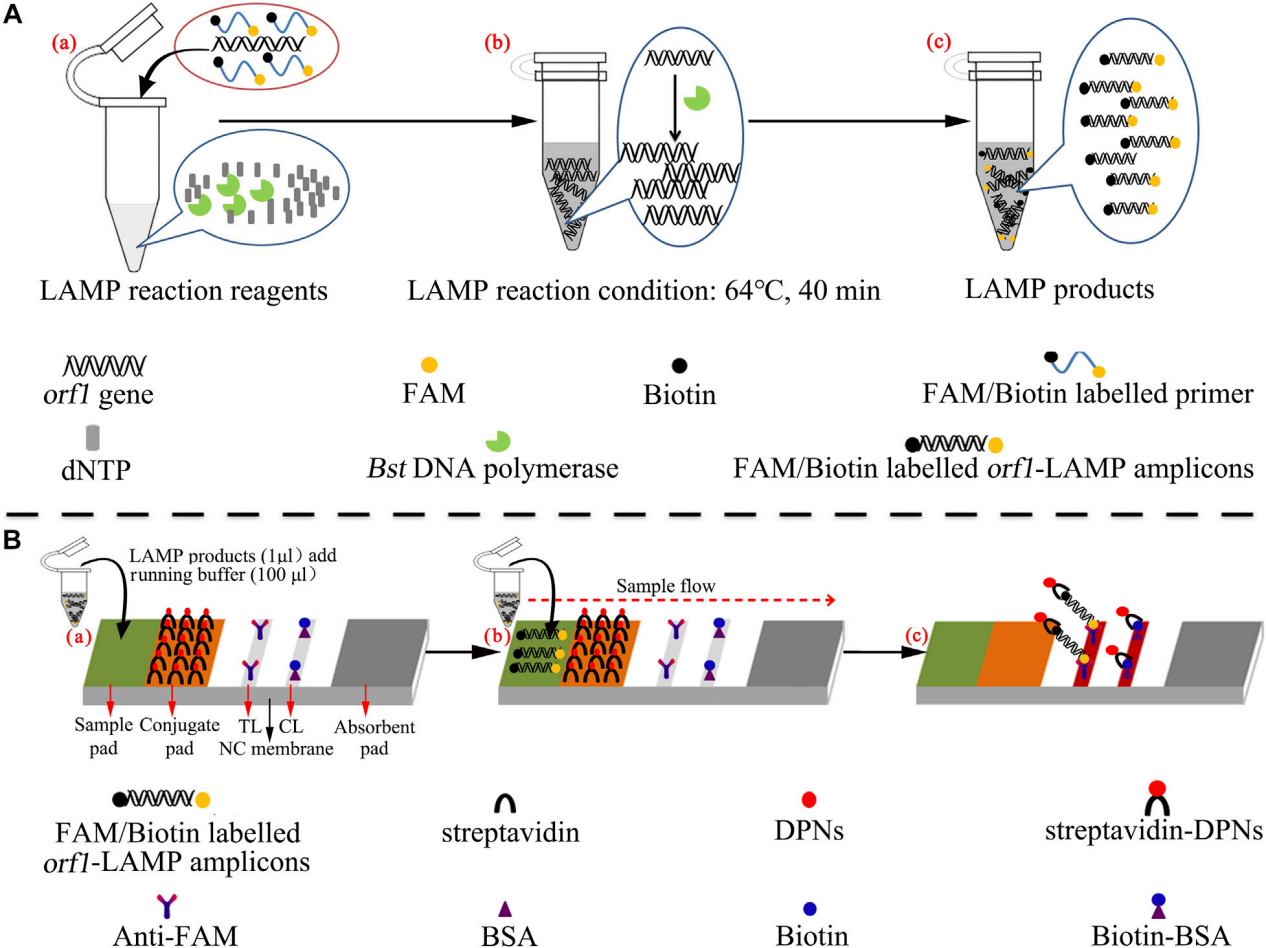
Mechanistic description of *N. gonorrhoeae*-loop-mediated isothermal amplification linked to a polymer nanoparticle–based biosensor (LAMP-PNB) assay. **(A)** Process of *N. gonorrhoeae*-LAMP. **(B)** Schematic illustration of the principle of the polymer nanoparticle–based biosensor assay for visualization of *N. gonorrhoeae*-LAMP products. A positive result for the orf1 gene means that the test line (TL) and control line (CL) appear simultaneously on the biosensor. Only a control line (CL) appearing on the biosensor indicates a negative result. dNTP: deoxynucleotide triphosphate; FAM: carboxyfluorescein; DPNs: dye (crimson red)–coated polymer nanoparticles; anti-FAM: rabbit anti-carboxyfluorescein antibody; BSA: bovine serum albumin; NC: nitrocellulose.

In the current study, the loop-mediated isothermal amplification linked with the polymer nanoparticle–based biosensor (LAMP-PNB) technique was devised firstly for simple, specific, sensitive, rapid, and visual identification of *N. gonorrhoeae* by targeting the *orf1* gene (Chaudhry and Saluja, 2002; Edwards et al., 2014), and it showed no homology with other microbial genomes in GenBank by BLAST searches. The principle of the *N. gonorrhoeae*-LAMP-PNB assay is illustrated in Figures 1A,B, and the detection performance was analyzed with pure cultures and clinical samples.

## Materials and Methods

### Materials and Reagents

Thayer-Martin (TM) chocolate agar plates were obtained from Autobio Biotechnology Co., Ltd. (Zhengzhou, China). Nucleic acid–releasing agents were obtained from Sansure Biotech Inc. (Changsha, China). Universal isothermal amplification kits and a colorimetric indicator (malachite green, MG) were purchased from HuiDeXin Bio-technology (Tianjin, China). Anti-FAM (rabbit anti-fluorescein antibody) and biotin BSA (biotinylated bovine serum albumin) were purchased from Abcam Co., Ltd. (Shanghai, China). Streptavidin dye–coated polymer nanoparticles (Crimson red) were obtained from Bangs Laboratories, Inc. (Indiana, United States). Polymer nanoparticle–based lateral flow biosensor (LFB) materials, including the sample pad, conjugate pad, absorbent pad, nitrocellulose (NC) membrane, and backing card, were obtained from HuiDeXin Bio-technology (Tianjin, China). *N. gonorrhoeae* PCR diagnosis kits were purchased from DaAn Gene Co., Ltd. (Guangzhou, China).

### Preparation of Clinical Samples and Bacterial Strains

In the current study, a total of 86 clinical samples were collected from suspected *N. gonorrhoeae*–infected patients at Hangzhou Women’s Hospital (Hangzhou, China) between June 2020 and February 2021. Two genital secretion samples were collected from each suspected *N. gonorrhoeae*–infected patient with sterile swabs. One sample was used for traditional cultivation detection and the other for genomic DNA preparation. The *N. gonorrhoeae* reference strain (ATCC 49926) and clinical samples were cultured on Thayer-Martin (TM) chocolate agar plates (Autobio, Zhengzhou, China) at 37°C in 5% CO_2_ for 2 days. Genomic DNA templates were obtained using nucleic acid–releasing agents (Sansure Biotech, Changsha, China) in accordance with the manufacturer’s instructions. In brief, 10 μl of samples were added into 10 μl of nucleic acid–releasing agents and incubated at room temperature (∼25°C) for 10 min to release nucleic acid. And then, the genomic DNA was stored at −20°C before use. The concentration was identified with NanoDrop 2000 (Thermo, United States) at A260/280, and the corresponding genome copy number was calculated from the weight of the *N. gonorrhoeae* genome. One copy of the *N. gonorrhoeae* genome is 2.45 fg (2.2 × 10^6^ bp (genomic DNA length) × 665 Da/bp×1.67 × 10^−24^ g/Da) (Dempsey et al., 1991; Geraats-Peters et al., 2005; Edwards et al., 2014).

### *N. gonorrhoeae*-LAMP Primer Design

**FIGURE 2:**
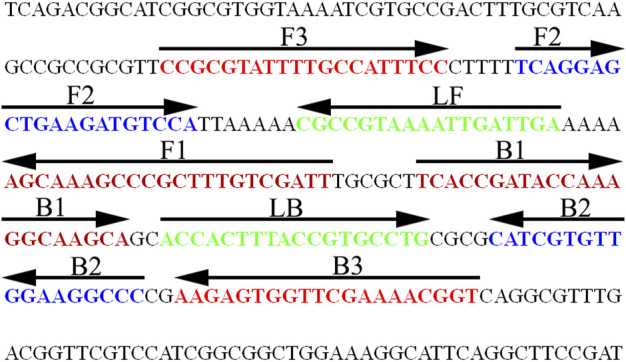
Nucleotide sequence and location of the *orf1* gene used to design the *N. gonorrhoeae*-LAMP primers. The part of nucleotide sequence of the sense strand of the *orf1* gene is shown in the diagram. The primer sequences are marked in different colors. Right arrows and left arrows indicate sense and complementary sequences, respectively.

A set of special LAMP primers, targeting on the *orf1* gene of *N. gonorrhoeae* (GenBank Accession No. M84113), was designed according to the LAMP principle. The LAMP primers consist of two outer primers (F3 and F3), two inner primers (FIP and BIP), and two loop primers (LF and LB). The specificity of *N. gonorrhoeae*-LAMP primers was analyzed using the Basic Local Alignment Search Tool (BLAST). Moreover, OligoAnalyzer online software (V3.1; Integrated DNA Technologies, Coralville, IA, United States) was employed for primer, dimer, and secondary structure investigation. The details of *N. gonorrhoeae*-LAMP primers’ location and sequences are shown in Figure 2 and Table 1, respectively.

**TABLE 1:** *N. gonorrhoeae*-LAMP primers used in the present study.

Primer name	Sequence and modifications	Length	Gene
F3	5ʹ-CCG​CGT​ATT​TTG​CCA​TTT​CC-3ʹ	20 nt	*orf1*
B3	5ʹ-ACC​GTT​TTC​GAA​CCA​CTC​TT-3ʹ	20 nt	
FIP*	5ʹ-FAM-AATCGACAAAGCGGGCTTTGCTTCAGGAGCTGAAGATGTCCA-3ʹ	42 mer	
BIP	5ʹ-TCA​CCG​ATA​CCA​AAG​GCA​AGC​AGG​GCC​TTC​CAA​CAC​GAT​G-3ʹ	40 mer	
LF*	5ʹ-biotin-TCAATCAATTTTACGGCG-3ʹ	18 nt	
LB	5ʹ-ACC​ACT​TTA​CCG​TGC​CTG-3ʹ	18 nt	

FIP*, 5ʹ-labeled with FAM when used with the LAMP-PNB assay; LF*, 5ʹ-labeled with biotin when used in the LAMP-PNB assay. Abbreviations: FAM, carboxyfluorescein; nt, nucleotide; mer, monomeric unit.

### Polymer Nanoparticle–Based Biosensor Preparation

The polymer nanoparticle–based biosensor (PNB) is illustrated in Figure 1B. In brief, the PNB consisted of four components: a sample pad, conjugate pad, nitrocellulose (NC) membrane, and absorbent pad. These components were assembled uniformly on a backing card. The capture reagents, including anti-FAM and biotin BSA, were immobilized by physical adsorption on the reaction regions. Then, anti-FAM was immobilized at test line 1 (TL1) (*N. gonorrhoeae*), while biotin BSA was immobilized at the control line (CL); each line was separated by 5 mm, and the detector reagents (streptavidin dye–coated polymer nanoparticles, SA-PNPs) were sprayed onto the conjugate pad. Therefore, the biosensor can detect two targets, including *N. gonorrhoeae*-LAMP amplicons and a chromatography control. Finally, the assembled biosensors were preserved in a plastic box and dry-stored at room temperature before use.

### Loop-Mediated Isothermal Amplification Reaction and Detection

The standard *N. gonorrhoeae*-LAMP was conducted in a 25 μl reaction system, containing 1 μl of DNA template; 0.4 μM of each outer primer, F3 and B3; 1.6 μM of each inner primer, FIP* and BIP; 0.8 μM of each loop primer, LF* and LB; 1 μl (8 U) of *Bst* DNA polymerase; and 12.5 μl of 2× reaction buffer (40 mM of Tris-HCl (pH 8.8), 40 mM of KCl, 16 mM of MgSO_4_, 20 mM of (NH_4_)_2_SO_4_, 2 M of betaine, and 0.2% Tween-20) (HuiDeXin Bio-technology, Tianjin, China); then, double distilled water was added to the 25 μl reaction system. The mixtures were heated at 64°C for 1 h. Genomic DNA from non–*N. gonorrhoeae* strains, including *Chlamydia trachomatis* and *Neisseria meningitidis*, was used as a negative control (NC), and double distilled water (DW) was used as the template in the blank control (BC).

Real-time turbidity (LA-500), colorimetric indicator (malachite green, MG), and the polymer nanoparticle–based biosensor (PNB) were used to confirm the LAMP reaction and optimize the reaction conditions. For the real-time turbidity method, turbidity >0.1 was considered a positive outcome. For the colorimetric indicator, the color changed from colorless to light green in the reaction system, indicating positive amplification products, while the color remained colorless in negative results. For the polymer nanoparticle–based biosensor, the test line (TL) and control line (CL) appeared simultaneously, indicating positive outcomes, but in negative amplification, only the control line (CL) could be observed.

### Optimization of *N. gonorrhoeae*-LAMP Reaction Temperature

Reaction temperature is critical for efficient LAMP. In the current study, the reaction temperature was optimized ranging from 60 to 67°C (with 1°C intervals). The *N. gonorrhoeae*-LAMP products were analyzed using real-time turbidity (LA-500). Turbidity >0.1 was considered a positive outcome.

### Sensitivity of *N. gonorrhoeae*-Loop-Mediated Isothermal Amplification Linked With the Polymer Nanoparticle–Based Biosensor Assay

The limit of detection (LoD) of the *N. gonorrhoeae*-LAMP-PNB assay was analyzed through the *N. gonorrhoeae* genomic DNA with 10-fold serial dilution from 5.0 × 10^4^ to 5.0 × 10^−1^ copies using double distilled water (DW). The *N. gonorrhoeae*-LAMP reactions were carried out as described above, and the results were identified using the colorimetric indicator (MG) and PNB methods, respectively. Simultaneously, the diluted concentration of the *N. gonorrhoeae* genome DNA was used for confirming the optimal isothermal time of the *N. gonorrhoeae*-LAMP-PNB assay.

In order to further confirm the sensitivity of the *N. gonorrhoeae*-LAMP-PNB assay, the *N. gonorrhoeae* strains were added into 100 μl of healthy volunteer urine, which made the final concentrations of *N. gonorrhoeae* strains range from 5.0 × 10^4^ to 5.0 × 10^−1^ copies/μl, respectively. The samples were centrifuged at 12,000 × g for 5 min (4°C), and the collected bacteria were suspended in 100 μl of nucleic acid–releasing agents (Sansure Biotech, Changsha, China) and incubated at room temperature (∼25°C) for 10 min for releasing nucleic acid. And then, 1 μl of genomic DNA was added for the *N. gonorrhoeae*-LAMP-PNB assay. The results were identified using MG and PNB methods, respectively. Each examination was conducted independently at least three times.

### Specificity of *N. gonorrhoeae*-Loop-Mediated Isothermal Amplification Linked With the Polymer Nanoparticle–Based Biosensor Assay

The analytical specificity of the *N. gonorrhoeae*-LAMP-PNB assay was evaluated by comparing *N. gonorrhoeae* genomic DNA with the templates from various bacteria, fungi, and viruses (Table 2). All examinations were confirmed in triplicate.

**TABLE 2:** Pathogens used in the current study.

No.	Pathogen	Source of pathogens^a^	No. of strains	*N. gonorrhoeae*-LAMP result^b^
1	*N. gonorrhoeae* (reference strain)	ATCC 49926	1	P
2	*N. gonorrhoeae* (clinical samples)	Hangzhou Women’s Hospital	18	P
3	*Chlamydia trachomatis*	Hangzhou Women’s Hospital	1	N
4	*Neisseria meningitidis*	Hangzhou Women’s Hospital	1	N
5	*Pseudomonas aeruginosa*	2^nd^ GZUTCM	1	N
6	*Leptospira interrogans*	Zhejiang Hospital	1	N
7	*Hemophilus influenzae*	ATCC 49247	1	N
8	*Candida glabrata*	2^nd^ GZUTCM	1	N
9	*Mycobacterium tuberculosis*	GZCCL	1	N
10	*Streptococcus pyogenes*	2^nd^ GZUTCM	1	N
11	*Mycoplasma pneumoniae*	Hangzhou Women’s Hospital	1	N
12	*Acinetobacter baumannii*	2^nd^ GZUTCM	1	N
13	*Shigella flexneri*	Zhejiang Hospital	1	N
14	*Staphylococcus aureus*	2^nd^ GZUTCM	1	N
15	Enteropathogenic *Escherichia coli*	Zhejiang Hospital	1	N
16	*Cryptococcus neoformans*	ATCC 14053	1	N
17	*Bordetella pertussis*	GZCCL	1	N
18	*Klebsiella pneumoniae*	GZCCL	1	N
19	Adenoviruses	Hangzhou Women’s Hospital	1	N
20	Respiratory syncytial virus type A	Hangzhou Women’s Hospital	1	N
21	Human rhinovirus	Hangzhou Women’s Hospital	1	N
22	*Hemophilus parainfluenzae*	GZCCL	1	N

a ATCC, American Type Culture Collection; 2^nd^ GZUTCM, Second Affiliated Hospital, Guizhou University of Traditional Chinese Medicine; GZCCL, Guizhou Provincial Center for Clinical Laboratory.

^b^P, positive; N, negative.

Confirming the Feasibility of the *N. gonorrhoeae*-LAMP-PNB Assay Using Clinical Samples

To verify the feasibility of *N. gonorrhoeae*-LAMP-PNB detection, 86 suspected *N. gonorrhoeae*–infected genital secretion samples were collected from Hangzhou Women’s Hospital (Hangzhou, China). And then, all of these samples were detected simultaneously using traditional culture, qPCR, and the LAMP-PNB assay, respectively. The culture detection was performed as previously described. The qPCR diagnosis was carried out using Gonococcus Nucleic Acid Assay Kits (DaAn Gene Co., Ltd., China) (Cat. #DA-D053), and the detection was performed with the Applied Biosystems™ 7500 Real-Time PCR System (Life Technologies, Singapore). According to the manufacturer’s instructions, concentrations of *N. gonorrhoeae* less than 500 copies will be considered negative results (the LoD of the qPCR assay is 500 copies per test). The *N. gonorrhoeae*-LAMP-PNB operation was performed as described above. The *N. gonorrhoeae* cultivation, qPCR, and LAMP-PNB assay were performed in a biosafety level 2 laboratory, as detailed in the WHO Laboratory biosafety manual, 3^rd^ edition.

### Statistical Analysis

The performance of LAMP-PNB was evaluated using bacteriologic results and clinical evidence as the composite reference standard. The χ^2^ test was used to compare sensitivity differences between cultivation, qPCR, and LAMP-PNB. SPSS 23.0 software was used to perform statistical analysis, and *p* < 0.05 was considered statistically significant.

## Results

### Confirmation and Detection of *N. gonorrhoeae*-LAMP Products

**FIGURE 3:**
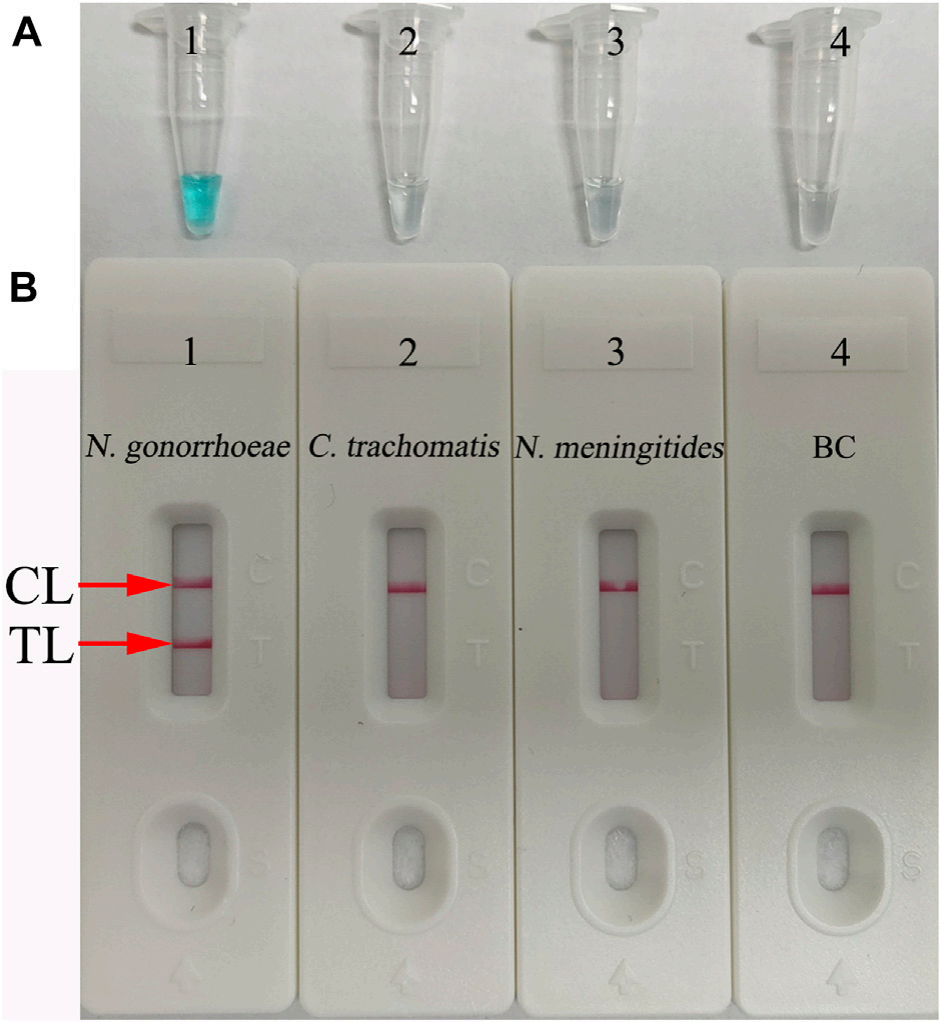
Determination and confirmation of the *N. gonorrhoeae*-LAMP products. The *N. gonorrhoeae*-LAMP products were analyzed with the colorimetric indicator **(A)** and polymer nanoparticle–based biosensor **(B)**. Tube 1/Biosensor 1: positive results of the *N. gonorrhoeae* reference strain (ATCC 49926); Tube 2/Biosensor 2: negative results of *Chlamydia trachomatis*; Tube 3/Biosensor 3: negative results of *Neisseria meningitidis*; Tube 4/Biosensor 4: blank control (distilled water). TL: test line; CL: control line.

To confirm the validity of the *N. gonorrhoeae*-LAMP reaction system, LAMP products were monitored using the colorimetric indicator (MG) and polymer nanoparticle–based biosensor (PNB). The reaction tubes with positive results of the *N. gonorrhoeae*-LAMP assay were visualized in light green, while the reaction tubes of negative and blank controls remained colorless (Figure 3A). Using the PNB, the TL and CL were observed in the detection region, indicating positive results. Only the CL appeared on the analysis area of the PNB for negative and blank controls (Figure 3B). Therefore, these results indicated that the *N. gonorrhoeae*-LAMP-PNB assay using *orf1*-LAMP primers designed in the current study was valid for the reliable and rapid detection of *N. gonorrhoeae*.

### Optimization of *N. gonorrhoeae*-LAMP Temperature

**FIGURE 4:**
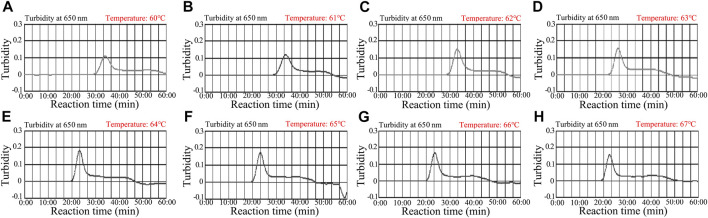
Optimization of temperature for the *N. gonorrhoeae*-LAMP reaction. The *N. gonorrhoeae*-LAMP reactions were monitored using real-time turbidity (LA-500), and the corresponding curves of amplicons are shown in the charts. The threshold value was 0.1, and turbidity >0.1 was considered to show positive amplification. Eight kinetic graphs **(A–H)** were obtained at different temperatures (60–67°C, 1°C intervals) with 5.0 × 10^3^ copies of genomic DNA from *N. gonorrhoeae* isolates (ATCC 49926). The optimal LAMP temperature was selected according to higher turbidity. The results indicated that the temperature of 64°C **(E)** showed robust amplification.

Reaction temperature is crucial for LAMP. In the current study, the reaction temperature of the *N. gonorrhoeae*-LAMP system was tested ranging from 60 to 67°C (with 1°C intervals) with 5.0 × 10^3^ copies of genomic DNA extracted from the reference strain (ATCC 49926). The *N. gonorrhoeae*-LAMP protocol was carried out as described above, and the results were monitored using real-time turbidity (LA-500). The results presented that the *N. gonorrhoeae*-LAMP was amplified faster in the temperature range from 64 to 67°C according to kinetic data (Figure 4). Hence, 64°C was considered the optimal amplification temperature for the subsequent *N. gonorrhoeae*-LAMP-PNB assay in this study.

### Sensitivity of the *N. gonorrhoeae*-LAMP-PNB Assay

**FIGURE 5:**
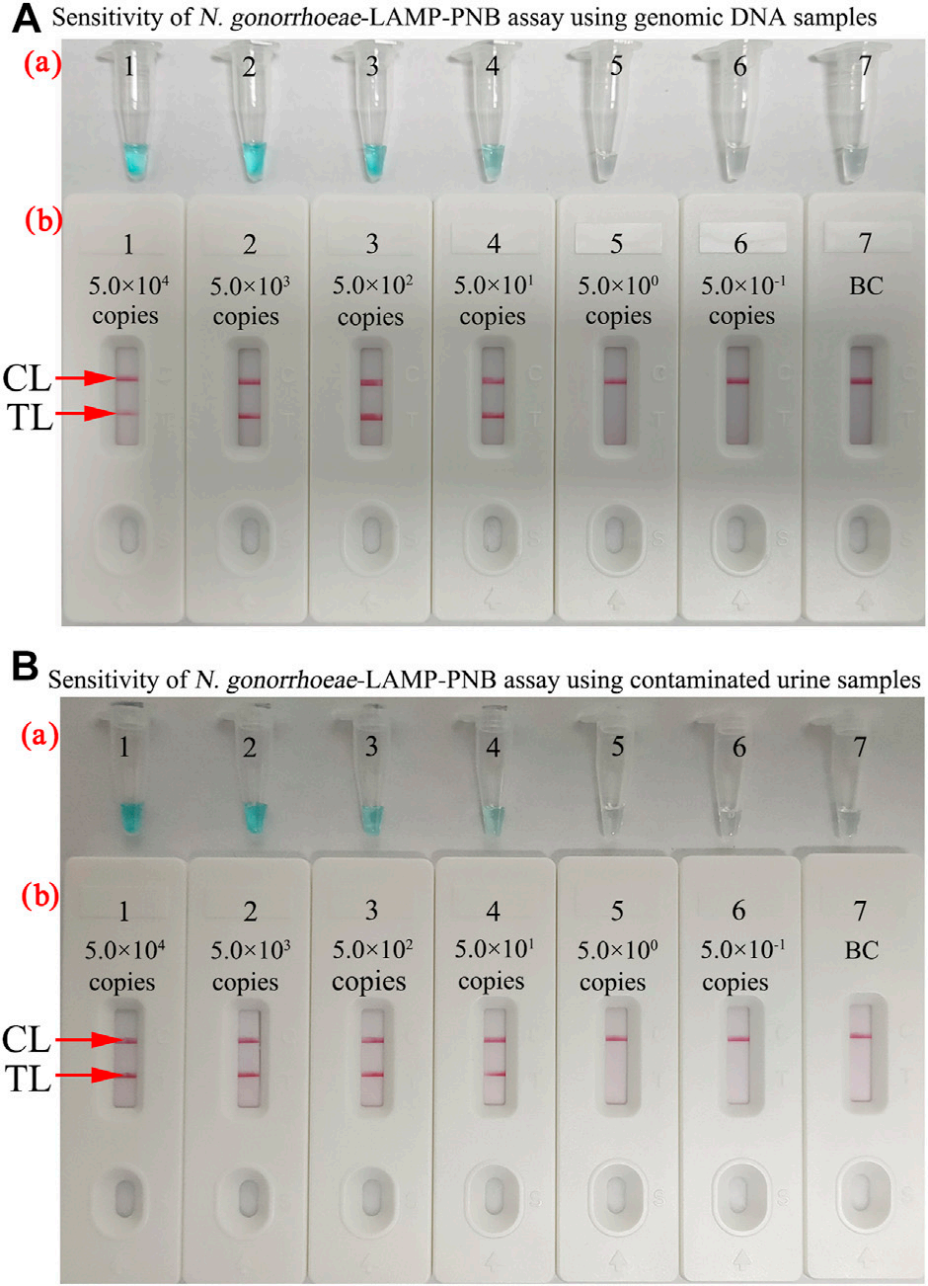
Sensitivity of the *N. gonorrhoeae*-LAMP-PNB assay. **(A)** Sensitivity of the *N. gonorrhoeae*-LAMP-PNB assay using diluted genomic DNA templates. MG (a) and PNB (b) were applied for reporting the *N. gonorrhoeae*-LAMP results. Tubes a1–a7 (Biosensors b1–b7) represent the genomic DNA levels of 5.0 × 10^4^ copies, 5.0 × 10^3^ copies, 5.0 × 10^2^ copies, 5.0 × 10^1^ copies, 5.0 × 10° copies, and 5.0 × 10^−1^ copies per reaction and blank control (DW), respectively. **(B)** Sensitivity of the *N. gonorrhoeae*-LAMP-PNB assay using contaminated urine samples. Each healthy volunteer’s urine samples contained different concentrations of *N. gonorrhoeae* strains (ranging from 5.0 × 10^4^ to 5.0 × 10^−1^ copies/μl). The *N. gonorrhoeae*-LAMP was performed as described above, and the results were analyzed with MG (a) and PNB (b), respectively. Tubes a1–a7 (Biosensors b1–b7) represent the *N. gonorrhoeae* strain concentrations of 5.0 × 10^4^ copies, 5.0 × 10^3^ copies, 5.0 × 10^2^ copies, 5.0 × 10^1^ copies, 5.0 × 10° copies, and 5.0 × 10^−1^ copies per reaction and blank control (DW), respectively. The results suggested that the LoD of the *N. gonorrhoeae*-LAMP-PNB assay was 5.0 × 10^1^ copies per test. CL: control line; TL: test line.

The sensitivity of the *N. gonorrhoeae*-LAMP-PNB assay was determined simultaneously with serial dilutions of genomic DNA and strains ranging from 5.0×10^4^ to 5.0 × 10^−1^ copies. The *N. gonorrhoeae*-LAMP protocol was performed as described above, and the products were verified with MG and the PNB. As shown in Figures 5A,B, the limit of detection (LoD) of the *N. gonorrhoeae*-LAMP assay was 50 copies.

### Optimal Amplification Time for *N. gonorrhoeae*-LAMP-PNB Assay

**FIGURE 6:**
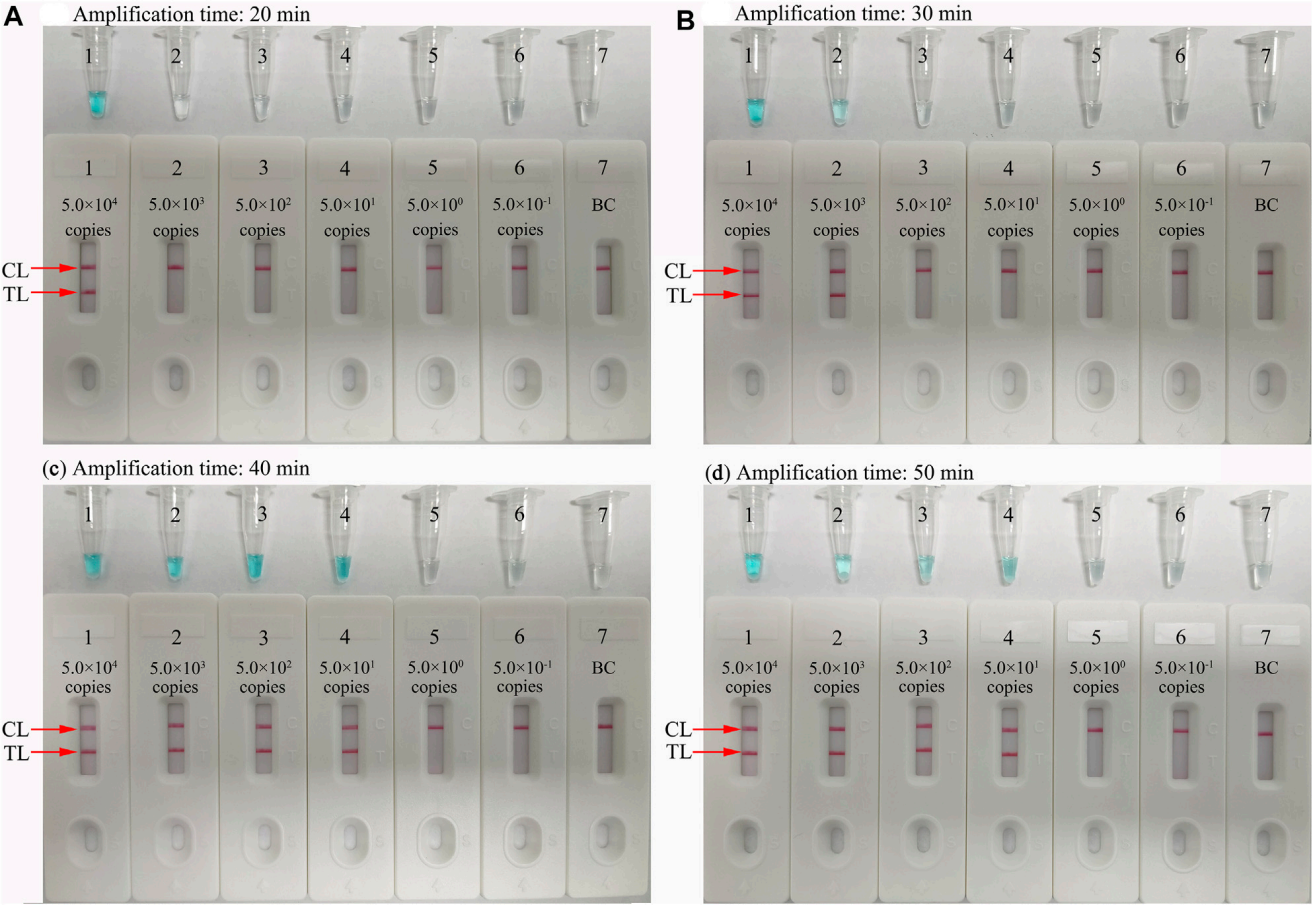
Optimal reaction time for the *N. gonorrhoeae*-LAMP-PNB assay. Four different amplification times (**A**, 20 min; **B**, 30 min; **C**, 40 min; **D**, 50 min) were tested at 64°C. Tubes/Biosensors 1, 2, 3, 4, 5, 6, and 7 represent *N. gonorrhoeae* (ATCC 49926) genomic DNA levels from 5.0 × 10^4^ to 5.0 × 10^−1^ copies per reaction and negative control (DW), respectively. The results indicated that the best sensitivity was detected when the reaction lasted for 40 min **(C)**. CL: control line; T: test line.

The optimal reaction time required for the *N. gonorrhoeae*-LAMP-PNB assay at the amplification stage was tested ranging from 30 to 60 min at the optimal reaction temperature (64°C). The results showed that the LoD of the template level (50 copies) displayed two crimson bands (CL and TL) when the isothermal amplification was conducted for 40 min (Figure 6). Hence, an *N. gonorrhoeae*-LAMP reaction time of 40 min was recommended for clinical sample detection. Thus, the whole detection procedure of the *N. gonorrhoeae*-LAMP-PNB technique, including genomic DNA template preparation (∼10 min), LAMP reaction (40 min), and resultant reporting (∼2 min), could be completed within 60 min.

### Specificity of the *N. gonorrhoeae*-LAMP-PNB Assay

**FIGURE 7:**
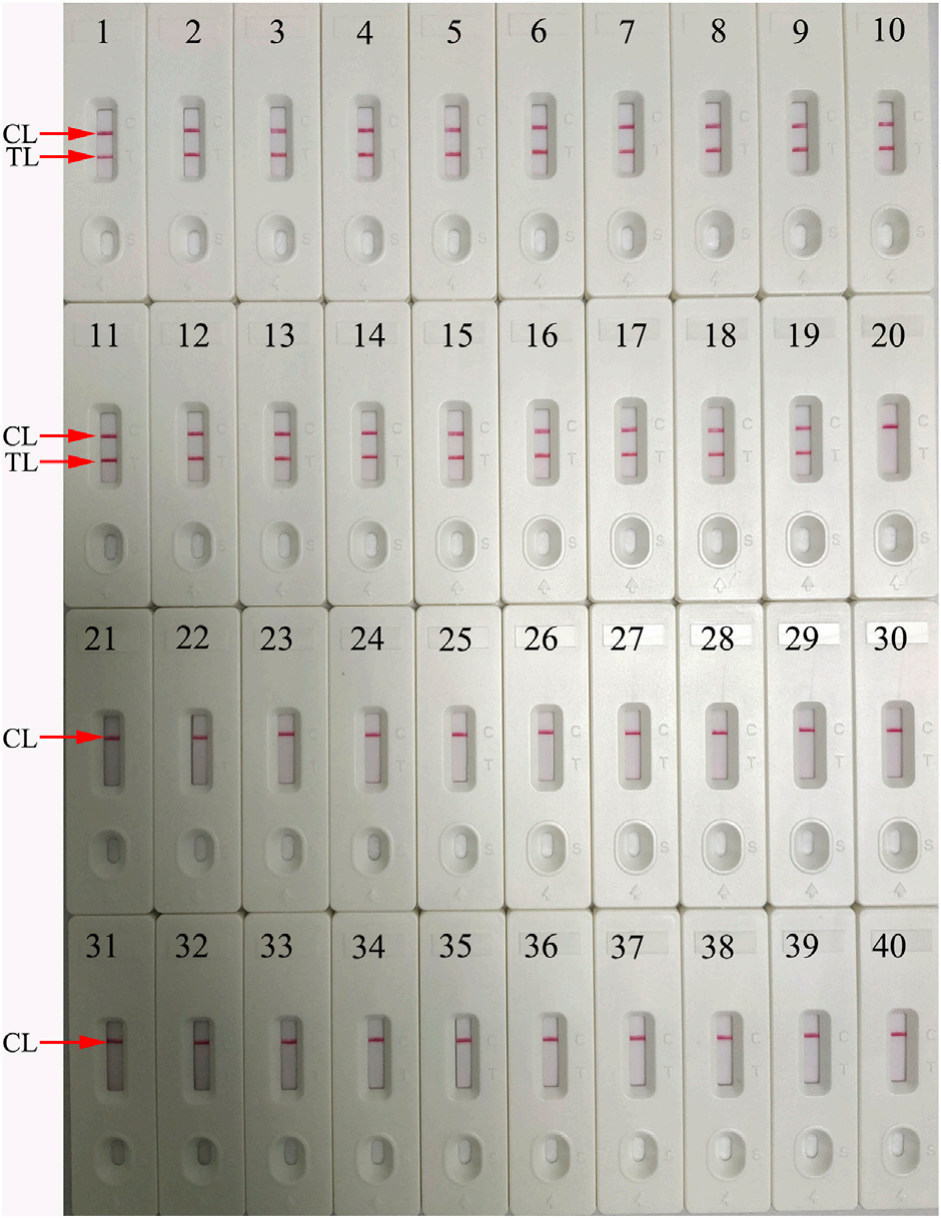
Analytical specificity of the *N. gonorrhoeae*-LAMP-PNB assay using different pathogens. The *N. gonorrhoeae*-LAMP-PNB assay was verified using different genomic RNA/DNA as templates. Biosensor 1, *N. gonorrhoeae* (ATCC 49926); Biosensor 2–19, *N. gonorrhoeae* (clinical samples); Biosensor 20, *Chlamydia trachomatis*; Biosensor 21, *Neisseria meningitidis*; Biosensor 22, *Pseudomonas aeruginosa*; Biosensor 23, *Leptospira interrogans*; Biosensor 24, *Hemophilus influenzae*; Biosensor 25, *Candida glabrata*; Biosensor 26, *Mycobacterium tuberculosis*; Biosensor 27, *Streptococcus pyogenes*; Biosensor 28, *Mycoplasma pneumoniae*; Biosensor 29, *Acinetobacter baumannii*; Biosensor 30, *Shigella flexneri*; Biosensor 31, *Staphylococcus aureus*; Biosensor 32, enteropathogenic *Escherichia coli*; Biosensor 33, *Cryptococcus neoformans*; Biosensor 34, *Bordetella pertussis*; Biosensor 35, *Klebsiella pneumoniae*; Biosensor 36, adenoviruses; Biosensor 37, respiratory syncytial virus type A; Biosensor 38, human rhinovirus; Biosensor 39, *Hemophilus parainfluenzae*; Biosensor 40, blank control. CL: control line; TL: test line.

The specificity of the *N. gonorrhoeae*-LAMP-PNB assay was tested with the *N. gonorrhoeae* reference strain (ATCC 49926), 18 *N. gonorrhoeae*–positive clinical samples, and 20 non–*N. gonorrhoeae* bacteria, fungi, and viruses (Table 2). The genomic DNA extracted from *N. gonorrhoeae* samples presented positive outcomes, while other target templates from non–*N. gonorrhoeae* pathogens were not detected (Figure 7). These results suggested that the LAMP-PNB assay presents very good specificity for *N. gonorrhoeae* identification.

### Evaluation of the *N. gonorrhoeae*-LAMP-PNB Assay in Clinical Samples

A total of 86 suspected *N. gonorrhoeae*–infected genital secretion samples, which were collected from Hangzhou Women’s Hospital (Hangzhou, China), were tested for verifying the feasibility of the *N. gonorrhoeae*-LAMP-PNB assay. All of these samples were simultaneously analyzed with biotechnical culture, qPCR, and the LAMP-PNB assay. The results showed that 58 of 86 clinical samples tested as *N. gonorrhoeae*–positive through conventional culture methods. The *N. gonorrhoeae*-LAMP-PNB assay results were consistent with the traditional cultivation testing outcomes. Using qPCR only found 55 positive outcomes (Table 3). These data suggested that our *N. gonorrhoeae*-LAMP-PNB assay established in the current study is more powerful for identifying gonorrhea patients, especially those at the initial stage of *N. gonorrhoeae* infection.

**TABLE 3:** Comparison of conventional culture, qPCR, and LAMP-PNB methods for testing *N. gonorrhoeae* in clinical samples.

Detection assay	Results	Gold standard method (cultivation)		True positive rate (%)	True negative rate (%)
		**+**	**−**
qPCR	+	55	0	94.8^a^	100
	−	3	28		
LAMP-PNB	+	58	0	100	100
	−	0	28		

a Statistically significant (*p* < 0.05) when compared with LAMP-PNB.

## Discussion

Sexually transmitted gonorrhea, caused by *N. gonorrhoeae*, remains a serious global public health challenge. More importantly, the overwhelming majority of gonococcal infections are in low- and middle-income regions (Chemaitelly et al., 2019). Hence, it is vital to devise adequate reliable, simple, cost-saving, and easy-to-use diagnosis assays for detecting *N. gonorrhoeae* infection. In the current study, loop-mediated isothermal amplification linked with the polymer nanoparticle–based biosensor (LAMP-PNB) was devised and successfully used for visual and rapid detection of *N. gonorrhoeae* strains.

An ideal laboratory diagnostic technique for identification of *N. gonorrhoeae* should be specific, sensitive, rapid, and affordable (Meyer and Buder, 2020). Traditionally, direction microscopy may produce rapid outcomes but lacks sensitivity in many cases, especially for asymptomatic patients; the load of gonococci to be detected is usually too low. Besides, other *Neisseria* species have a similar morphology to *N. gonorrhoeae*. Therefore, the method is not recommended (World Health Organization, 2013; Mensforth et al., 2018). Bacterial cultivation was considered a “golden standard” for specific and sensitive identification of *N. gonorrhoeae* isolates. But gonococci require demanding and fastidious growth conditions and often obtain frustrating results (Visser et al., 2020). Moreover, the culture process is time-consuming. In recent years, nucleic acid amplification technologies (NAATs) were considered the primary tests for identification of gonococci in clinical practice (Fifer et al., 2019). However, their use in low- and middle-income regions is greatly limited due to requiring expensive instruments and trained experts. In this study, the *N. gonorrhoeae*-LAMP-PNB assay merges isothermal amplification with a polymer nanoparticle–based biosensor, which is achieved with extremely simple instruments, such as a water bath, a heating block, or even a thermos cup that can keep a constant temperature (64°C) for 40 min. More importantly, the biosensor provides an easy-to-use platform, which could visually and objectively detect the *N. gonorrhoeae*-LAMP products. The whole detection process, including genomic DNA template preparation (∼10 min), LAMP reaction (40 min), and product reporting (∼2 min), could be completed within 60 min. Therefore, the *N. gonorrhoeae*-LAMP-PNB assay is a rapid, economical, and simple assay for identification of gonococci in clinical settings, especially in resource-constrained regions.

Loop-mediated isothermal amplification (LAMP), as a reliable, sensitive, and rapid assay with low-cost equipment, was devised in 2000 and has been widely applied to detect many pathogens (Notomi et al., 2000; Li et al., 2019; Shete et al., 2019; Zhu et al., 2020). It involves only *Bst* DNA polymerase, with strand displacement capability and isothermal amplification, operating at a consistent temperature (between 60 and 68°C) throughout the reaction. The isothermal amplification of specific nucleic acid sequences is achieved by employing a set of four (or six) specific primers spanning six (or eight) distinct regions of the target gene (Li et al., 2017; Zhang et al., 2019). In the current study, a set of *N. gonorrhoeae*-LAMP primers was specifically designed to identify eight regions of the target fragment (Figure 2). The specificity of the *N. gonorrhoeae*-LAMP-PNB assay was powerfully confirmed with nucleic acid extracted from *N. gonorrhoeae* clinical samples and other pathogens. The positive results were observed from positive control and gonococci clinical samples, but non–*N. gonorrhoeae* agents showed negative results (Figure 7). Thus, our approach displayed a high level of specificity for identification of the gonococci agent. In addition to its excellent specificity, the newly developed *N. gonorrhoeae*-LAMP-PNB assay was able to detect pathogens in as low as 50 copies (Figure 5), which is sufficient for the diagnosis of gonorrhea. For detection of clinical samples, our *N. gonorrhoeae*-LAMP-PNB assay was highly sensitive compared to the qPCR method (Table 3), and the assay results were completely consistent with the traditional cultivation testing outcomes.

In this report, polymer nanoparticles, as a carrier material, were used for preparation of the nanoparticle-based biosensor (PNB). Owing to high surface-to-volume ratio, high adsorption and reactive capacity, good biocompatibility, and their easy-to-synthesize and -manipulate nature, polymer nanoparticles are the most appropriate nanomaterial used as a biosensor (Quesada-González and Merkoçi, 2015; Wang et al., 2017; Anniebell and Gopinath, 2018). The PNB could visually detect the *N. gonorrhoeae*-LAMP products for labeling with anti-FAM and BSA biotin on the PNB strips. The two crimson red bands—test line (TL) and control line (CL)—appeared in the PNB strip indicating positive results, while the negative outcomes only observed the control line (CL) in the biosensor. Although the colorimetric indicator (MG reagent) and real-time turbidity could identify *N. gonorrhoeae*-LAMP products, the former is ambiguous when the concentration of the amplicon is low and the latter method is laborious and has specific high-cost equipment. The PNB is simple and easy-to use, and the cost of the PNB designed in our study is estimated to be $2 USD per test. Hence, we calculate that the total cost of each test, including genomic DNA template preparation (∼€ 0.9 euros), LAMP (∼€2.9 euros), and LFB identification (∼€1.7 euros), is estimated to be € 5.5 euros, which is cheaper than PCR-based techniques. More importantly, the *N. gonorrhoeae*-LAMP-PNB assay has great potential to develop a point-of-care (POC) testing method for identifying gonorrhea-suspected patients in clinical settings, especially in economically impoverished regions of the world.

## Conclusion

In the current study, a reliable, rapid, and low-cost *N. gonorrhoeae*-LAMP-PNB technique targeting the *orf1* gene was successfully devised for assaying gonococci isolates. Our data demonstrated that the *N. gonorrhoeae*-LAMP-PNB was a highly sensitive and specific diagnostic method and could be used as an attractive laboratory tool for diagnosis of gonorrhea in clinical settings. The test does not rely on expensive apparatus and reagents, and the whole process could be completed within 60 min. Hence, the *N. gonorrhoeae*-LAMP-PNB assay could be considered an ideal method for the reliable and rapid detection of *N. gonorrhoeae* in clinical application, particularly in resource-constrained regions of the world.

## Data Availability

The original contributions presented in the study are included in the article/supplementary material, and further inquiries can be directed to the corresponding authors.

## Abbreviations

ATCC, American Type Culture Collection; BC, blank control; CL, control line; DW, distilled water; FAM, carboxyfluorescein; GZCCL, Guizhou Provincial Center for Clinical Laboratory; LAMP, loop-mediated isothermal amplification; LAMP-PNB, loop-mediated isothermal amplification linked to a polymer nanoparticle–based biosensor; LoD, limit of detection; MG, malachite green; mer, monomeric unit; NC, negative control; nt, nucleotide; PCR, polymerase chain reaction; PNB, polymer nanoparticle–based biosensor; TL, test line; WHO, World Health Organization; ZJH, Zhejiang Hospital; 2^nd^ GZUTCM, Second Affiliated Hospital, Guizhou University of Traditional Chinese Medicine.
